# Adsorption Performance of Modified Graphite from Synthetic Dyes Solutions

**DOI:** 10.3390/ma17174349

**Published:** 2024-09-02

**Authors:** Yi Xin, Youyu Bai, Xiaowen Wu, Dingyi Zhang, Weihua Ao, Minghao Fang, Zhaohui Huang, Yanbin Yao

**Affiliations:** 1 Engineering Research Center of Ministry of Education for Geological Carbon Storage and Low Carbon Utilization of Resources, Beijing Key Laboratory of Materials Utilization of Nonmetallic Minerals and Solid Wastes, National Laboratory of Mineral Materials, School of Materials Science and Technology, China University of Geosciences, Beijing 100083, China; xinyi_cugb@163.com (Y.X.);; 2 School of Science, China University of Geosciences, Beijing 100083, China; zhangdy97@163.com; 3 School of Energy Resources, China University of Geosciences, Beijing 100083, China

**Keywords:** adsorption, coal-based graphite, dyeing wastewater, high-temperature treatment

## Abstract

Due to the severe harmful impacts of industrial dyeing wastewater on ecosystems and human health, proper treatment is crucial. Herein, the use of modified graphite as an adsorbent for dyeing wastewater treatment was investigated in this study. The graphite was oxidized and intercalated using a phosphoric acid–nitric acid–potassium permanganate system and then thermally treated at high temperatures to optimize its structure. By adjusting the thermal treatment temperature, the graphite adsorbent with varying porosity was obtained. The optimized graphite demonstrated significant improvement in adsorption performance for dyes and organic compounds, achieving a removal rate of over 85% for methylene blue (MB) dye. The optimal adsorption performance is achieved with a 1.6 mg modified graphite adsorbent at 60 °C under alkaline conditions for adsorbing 10 ppm MB. Adsorption kinetics and isotherm models were applied to elucidate the adsorption mechanisms. The results fit the Langmuir model, suggesting that monolayer homogeneous adsorption is favorable. Importantly, the results demonstrate that high-temperature treatment can significantly enhance the adsorption properties of coal-based graphite, supporting its application in dyeing wastewater treatment.

## 1. Introduction

Untreated dyeing wastewater discharged directly into water bodies can cause severe water pollution, posing significant threats to the environment and human health. The treatment of dyeing wastewater, particularly from industrial sources, poses significant environmental challenges due to its potential harmful effects on ecosystems and human health [1,2,3]. Dyeing wastewater contains a variety of toxic substances, including heavy metals, organic dyes, and other hazardous chemicals, making it difficult to treat using conventional methods [4]. Therefore, effective treatment methods are essential for meeting discharge standards and mitigating these risks. Additionally, the persistence and toxicity of many dyes necessitate advanced treatment solutions to efficiently remove these contaminants and prevent their adverse environmental impacts. In recent years, the development of efficient, cost-effective, and environmentally friendly methods for wastewater treatment has become a critical research focus [5,6].

Among various treatment methods, adsorption using carbon-based materials has shown great promise due to its simplicity, high efficiency, and environmental friendliness. In recent years, extensive research has been conducted on the application of carbon-based materials in wastewater adsorption [7,8,9]. Activated carbon, graphene, and expanded graphite are some of the widely studied adsorbents. Activated carbon is known for its high surface area and porosity, demonstrating excellent adsorption capacities. For instance, powdered activated carbon has shown an adsorption efficiency of up to 89% for synthetic dyes in wastewater under optimal conditions [10].

Coal-based graphite, a natural, abundant, and low-cost material, has gained attention for its potential in dyeing wastewater treatment due to its unique properties such as structural stability, chemical inertness, and high surface area [11]. The unique properties of coal-based graphite, such as its structural stability, chemical inertness, and high surface area, make it an attractive candidate for developing efficient adsorbents for wastewater treatment. Previous studies have demonstrated that modifying the surface chemistry and porosity of coal-based graphite enhances its adsorption capacity for various pollutants, making it a promising material for dye adsorption due to its high mechanical strength and large surface area [12]. Studies have reported that graphene oxide can achieve an adsorption capacity of 1095.70 mg/g for methylene blue, making it highly effective compared to traditional adsorbents [13]. In addition, expanded graphite, produced from natural flake graphite through oxidation and high-temperature expansion, also offers unique structural advantages [14]. The improved adsorption performances of expanded graphite in comparison with other commercial adsorbent materials indicate the best competence adsorbent material [15].

Especially, coal-based graphite, an abundant and low-cost material, has gained attention for its potential in wastewater treatment [16]. The unique properties of coal-based graphite, such as its structural stability, chemical inertness, and high surface area, make it an attractive candidate for developing efficient adsorbents for wastewater treatment. Previous studies have shown that modifying the surface chemistry and porosity of coal-based graphite can further improve its adsorption capacity for various pollutants [17,18]. Coal-based graphite is generally more cost-effective than MOFs. While MOFs are often synthesized using expensive metal ions and organic linkers, coal-based graphite is a natural, abundant, and relatively low-cost material, which makes it more economically viable for large-scale applications [19].

Herein, this study aims to investigate the structural regulation of high-temperature (joule heat treatment) treated coal-based graphite and its effectiveness in adsorbing dyes from dyeing wastewater. By oxidizing and intercalating coal-based graphite using a phosphoric acid–nitric acid–potassium permanganate system and subsequently optimizing its structure through high-temperature treatment, its adsorption properties can be significantly enhanced. Additionally, adsorption kinetics and isotherm models were used to analyze the adsorption mechanisms. The insights gained from this research will not only enhance our understanding of the adsorption mechanisms of coal-based graphite but also pave the way for its practical application in industrial wastewater management.

## 2. Experiments

### 2.1. Chemicals and Reagents

Coal-based graphite, sourced from Hunan Xinhua Mine and provided by Southern Graphite Research Institute Co., Ltd. (Hunan, China), was used in this experiment. Phosphoric acid (85%) was produced by Xilong Chemical Reagent Co., Ltd. (Shantou, China), and nitric acid (68%) and potassium permanganate (99.8%) were obtained from Beijing Chemical Factory Co., Ltd. (Beijing, China) Methylene blue (98.5%,CAS: 61-73-4) was supplied by Tianjin Guangfu Fine Chemical Research Institute (Tianjin, China), while methyl orange (CAS: 547-58-0) was produced by Xilong Chemical Reagent Co., Ltd. Additionally, concentrated hydrochloric acid was provided by Sinopharm Chemical Reagent Co., Ltd. (Shanghai, China), and sodium hydroxide was sourced from Aladdin Reagent Co., Ltd. (Shanghai, China). All the chemicals used in this study were of analytical grade and were used without any further purification.

### 2.2. Experimental Procedures

#### 2.2.1. Oxidation Intercalation of Coal-Based Graphite

The oxidation intercalation of coal-based graphite was carried out as follows: Phosphoric acid and nitric acid were mixed in a mass ratio of 5:3. Subsequently, 0.8 g of potassium permanganate was weighed and added to 2.0 g of coal-based graphite to initiate the oxidation reaction. For details, see Table 1. Then, 32 mL of the nitric acid–phosphoric acid mixture was added for the intercalation reaction, which was conducted at 30 °C for durations of 1 h and 2 h, respectively. The reaction mixture was then filtered and washed 3–4 times using a vacuum filtration apparatus, followed by drying in a constant-temperature oven. Samples treated chemically and by expansion were marked as modified graphite. Table 1 shows the raw material sample ratio and experimental conditions.

#### 2.2.2. High Temperature Control and Adsorption Property Test of Coal-Based Graphite

In this experiment, a vacuum tube furnace (VTF) and an ultrafast high-temperature furnace (UFTF) were used to treat coal-based graphite at different temperatures to regulate its structure and explore the optimal experimental conditions. Samples A and B of oxidized intercalated coal-based graphite were placed in the vacuum tube furnace, and argon gas was introduced. The temperature of the vacuum tube furnace was preset to 800 °C, and the samples were held at this temperature for 30 min. The ultrafast high-temperature furnace was then preset to 600 °C, 800 °C, 1000 °C, 1200 °C, and 1400 °C. Samples A and B were placed in the ultrafast high-temperature furnace, which was evacuated to create a vacuum and then operated with a holding time of 30 s at each preset temperature to obtain graphite treated at different temperatures. Table 2 shows the control temperature and equipment of samples.

The adsorption performance of the high-temperature regulated samples was tested by adding 10 mg of coal-based graphite samples treated under different conditions into centrifuge tubes containing 20 mL of 10 ppm MB solution and MO solution, respectively. The samples were shaken in a water bath shaker for 12 h to allow adsorption. Afterward, the samples were centrifuged using a high-speed centrifuge at 9000× *g* rpm for 3 min. The supernatant was then collected, and the absorbance of methylene blue was measured at the optimal absorption wavelength of 664 nm, while the absorbance of methyl orange was measured at the optimal absorption wavelength of 465 nm using a UV–Vis spectrophotometer.

#### 2.2.3. Calculation of Adsorption Capacity and Removal Rate

The preparation of organic dye solutions and the determination of standard curves are as follows. In this experiment, a TU1901 UV–Vis spectrophotometer was used to analyze the MB or MO content in simulated wastewater. By measuring the maximum absorbance of the dye at various concentrations, a calibration curve of absorbance versus concentration was plotted. To prepare the simulated MB or MO solution, 25 mg of MB or MO was accurately weighed and placed into a 250 mL volumetric flask. Ultra-pure water was added to dilute the solution up to the calibration mark, and the solution was shaken to ensure thorough dissolution, resulting in a 100 mg/L MB or MO solution.

**Figure 1:**
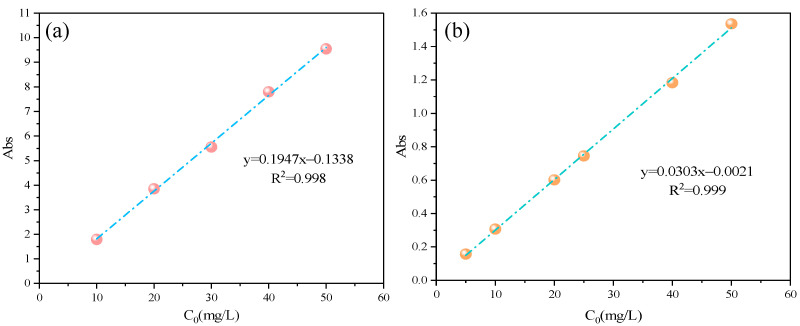
Standard curve of MB (**a**) and MO (**b**).

The standard curve of the MB and MO solution is shown in Figure 1a,b. The obtained fitting equations are y_MB_ = 0.1947X − 0.1338 and y_MO_ = 0.0303X − 0.0021 with correlation coefficients of R_MB_^2^ = 0.998 and R_MO_^2^ = 0.999. The absorbance of MB and MO in the supernatant was measured at the optimal absorption wavelength of 664 nm and 464 nm using a UV–Vis spectrophotometer. The corresponding concentration was calculated based on the MB or MO standard curve.

The adsorption capacity (q_t_) and removal rate (R) were calculated using Equations (1) and (2) [20,21], as follows:

(1) $$q_{t}=\frac{\left(C_{0}-C_{t}\right)\cdot V}{m}$$

(2) $$R=\frac{\left(C_{0}-C_{t}\right)}{C_{0}}\times 100\%$$

where *q_t_* represents the adsorption capacity at time *t*, mg/g; *R* represents the removal rate at time *t*; *C_0_* represents the initial concentration of the MB solution, mg/L; *C_t_* represents the concentration of the MB solution at time t, mg/L; *V* represents the total volume of the MB solution, L; and *m* represents the amount of adsorbent added, g.

### 2.3. Characterization

The samples were characterized by X-ray diffraction (XRD, D8 ADVANCE), ultrafast high-temperature furnace (E-shock 2022A, Particle Precision Instrument Co., Ltd., Jilin, China), and scanning electron microscopy (SEM, ZEISS SUPRA55, ZEISS, Oberkochen, Germany) methods. XRD measurement was carried out using Cu Kα radiation in the range of 5–80° (2θ) and λ = 1.5418 for phase identification and determination of the relative crystallinity.

## 3. Results and Discussion

### 3.1. Microstructure Analysis

**Figure 2:**
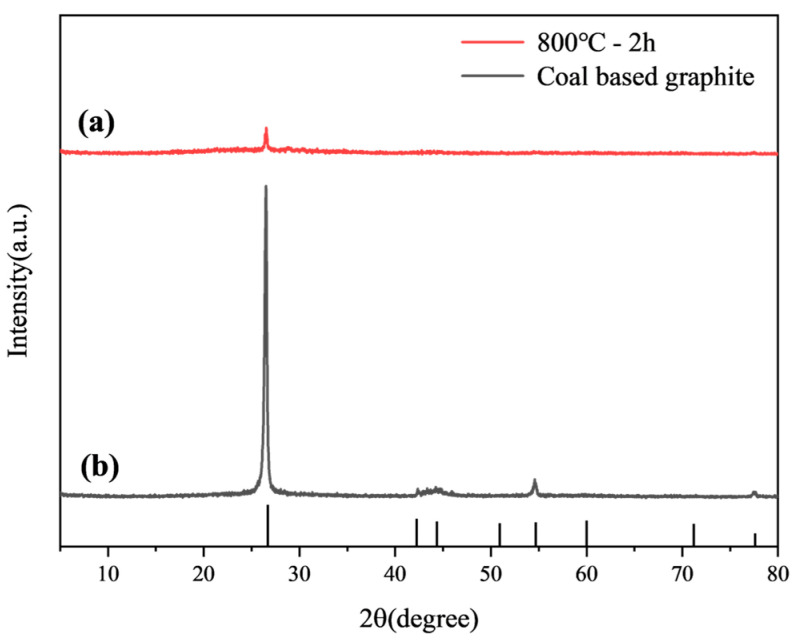
(**a**) XRD patterns of the optimal modified graphite and (**b**) raw coal-based graphite.

The XRD analysis of the raw coal-based graphite showed significant characteristic peaks. After treatment in a rapid high-temperature furnace, the XRD pattern showed reduced characteristic peaks indicating a decrease in crystallinity. The results are shown in Figure 2. As shown in Figure 2, the XRD analysis of the raw coal-based graphite indicates distinct characteristic peaks at 2θ = 26.4°, 42.4°, 44.45°, 54.60°, and 77.32°, corresponding to the (002), (100), (101), (004), and (110) crystal planes, respectively. After treatment with the ultrafast high-temperature furnace, the XRD pattern, as shown in Figure 2a, reveals that the characteristic peaks decrease, the crystal structure is disrupted, and the crystallinity is reduced.

**Figure 3:**
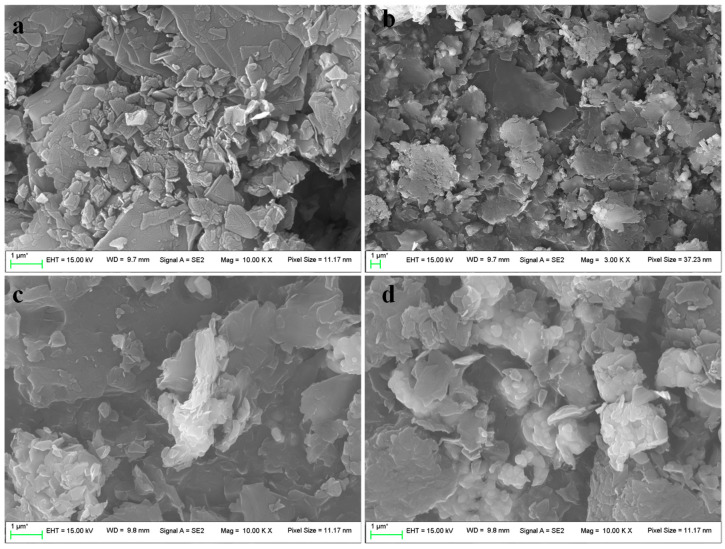
SEM spectrum of coal-based graphite without treatment (**a**,**b**); optimal porous carbon materials after treatment with the ultrafast high-temperature furnace (**c**,**d**). * represents an estimate or approximation.

As shown in Figure 3a,b, the raw coal-based graphite appears as irregular flakes with a relatively smooth surface and neat, regular edges. However, as shown in Figure 3c,d, the coal-based graphite treated with the ultrafast high-temperature furnace exhibits edge damage and curling due to the oxidation by the oxidizing agent. Additionally, the interlayer spacing has significantly increased, indicating that the intercalating compounds HNO_3_ and H_3_PO_4_ have entered the interlayers of the coal-based graphite, obtained a porous structure and endowed the graphite with a certain adsorption capacity [22].

### 3.2. Heating Process Effect of MB Adsorption

**Figure 4:**
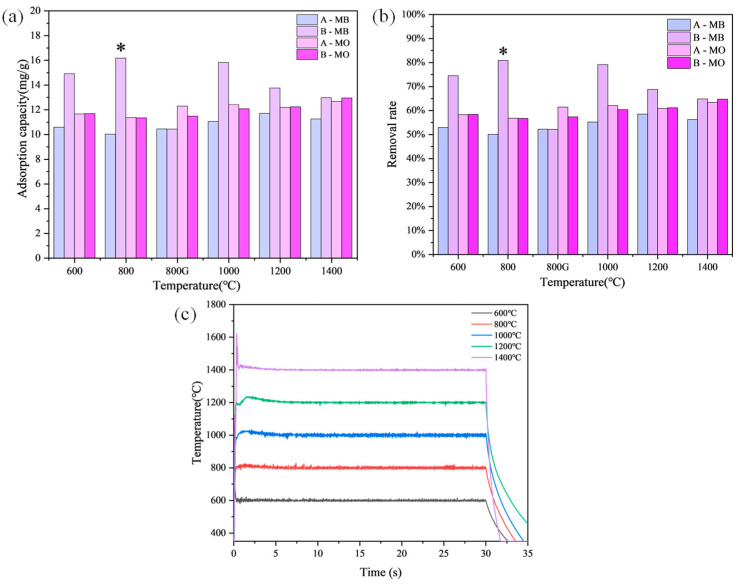
Adsorption capacities of different samples for MB and MO (**a**); Removal rates of different samples for MB and MO (**b**); The rising and falling temperature curves of the ultrafast high-temperature furnace (**c**). Notes: (**a**) G stands for reaction in a vacuum tube furnace; The asterisk (*) denotes the sample with the best performance among all samples.

After oxidation, intercalation, and high-temperature treatment, the modified graphite samples were evaluated for their adsorption capacity and removal rate. The results are shown in Figure 4. The adsorption experiments demonstrated that both Group A and Group B samples exhibited similar adsorption effects on MO, with removal rates around 60%. However, Group A samples showed significantly lower adsorption effects on MB compared to Group B samples. Specifically, the removal rate of MB for Group A was below 60%, while Group B samples had removal rates exceeding 70%. Notably, as seen in Figure 4b, the sample treated in a rapid high-temperature furnace at 800 °C (B-8) exhibited the best performance, achieving a removal rate of 80.85% for MB in Figure 4a. As seen in Figure 4c, the improved performance of the samples treated in the rapid high-temperature furnace can be attributed to the rapid heating rate, which facilitates the decomposition and gasification of graphite intercalation compounds more quickly, resulting in a better expansion effect. The rapid heating rate allows for the formation of a more porous structure, enhancing the adsorption capacity of the modified graphite [23]. Additionally, the short treatment time of 30 s in the rapid high-temperature furnace was sufficient to significantly improve the adsorption properties, compared to the longer treatment time of 30 s in the vacuum tube furnace. This indicates that both the heating rate and duration are crucial factors in determining the efficiency of the adsorption process.

Based on the results of high-temperature structural regulation and adsorption performance tests, the modified graphite prepared using the ultrafast high-temperature furnace at 800 °C was selected as the optimal adsorbent, with MB as the adsorption study target.

Furthermore, the presence of a more porous structure in the samples treated at 800 °C suggests that this temperature is optimal for achieving a balance between the thermal decomposition of intercalation compounds and the preservation of structural integrity. This optimal condition leads to enhanced adsorption sites and increased surface area, which are critical for the effective removal of dyes from aqueous solutions. The findings highlight the importance of optimizing thermal treatment parameters to maximize the performance of coal-based graphite in adsorption applications.

### 3.3. Effect of Adsorbent Amount

Different amounts of the optimal adsorbent were added to 5 mL of 10 mg/L MB solution, adjusting the initial amount of adsorbent to 2 mg, 4 mg, 6 mg, 8 mg, and 10 mg. The magnetic stirring speed was set at 550 rpm, the temperature was 25 °C, and the adsorption process lasted for 120 min to reach equilibrium. The supernatant from each corresponding vial was centrifuged at 4000× *g* rpm for 5 min, and the absorbance was measured to investigate the effect of varying the adsorbent dosage on the adsorption performance of MB.

**Figure 5:**
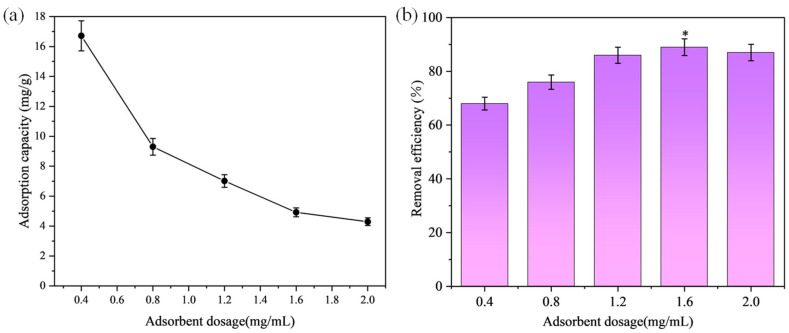
Effect of adsorbent amount on MB adsorption: (**a**) Adsorption capacity; (**b**) Removal efficiency. The asterisk (*) in the figure indicates the optimal amount of adsorbent added.

As shown in Figure 5, with the increase in the amount of modified graphite adsorbent, the removal rate of MB also increased. This is mainly because the increase in the amount of modified graphite adsorbent leads to an increase in the active sites available for MB adsorption in the solution, thus increasing the removal rate of MB. At an adsorbent dosage of 1.6 mg/mL, the adsorption effect was optimal, with a removal rate of approximately 90%. Considering both adsorption efficiency and cost, 1.6 mg/mL was selected as the optimal adsorbent dosage.

### 3.4. Effect of Temperature

**Figure 6:**
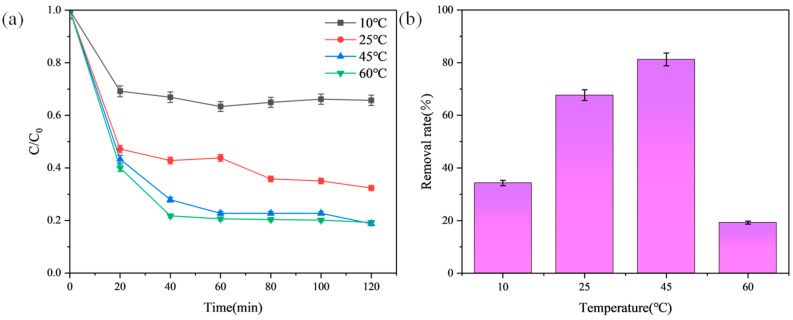
Effect of temperature on MB adsorption by the optimal adsorbent. (**a**) Adsorption curves of the adsorbent for MB at different temperatures; (**b**) Removal efficiency of MB by the adsorbent at different temperatures after 120 min.

An 8 mg prepared adsorbent was sequentially placed in small glass vials containing 5 mL of 50 mg/L MB solution and stirred at a constant speed of 550 rpm. Adsorption was conducted at 10 °C, 25 °C, 45 °C, and 60 °C. Supernatant samples were taken at intervals of 20 min, specifically at 20 min, 40 min, 60 min, 90 min, and 120 min. The supernatant was centrifuged at 4000 rpm for 5 min, and the absorbance was measured to explore the effect of temperature changes on MB adsorption performance [24]. Figure 6 shows the adsorption curves of MB on modified graphite at different temperatures.

During the adsorption process using modified graphite adsorbent, the removal rate of MB significantly increased with rising temperature. Typically, adsorption on solid adsorbents reaches equilibrium when the MB monolayer fully covers the adsorbent surface. Higher temperatures enhance adsorption capacity, possibly due to increased electrostatic interactions between MB and modified graphite. Water molecules also exhibit dipole–dipole interactions with modified graphite, occupying adsorption sites. Raising the temperature reduces adsorbent–solvent interactions, enhancing intermolecular forces between the adsorbent and the adsorbate. At lower temperatures, weaker electrostatic interactions between MB and modified graphite allow water to attach to adsorption sites. The increased adsorption capacity at higher temperatures indicates that the MB adsorption process is endothermic. Therefore, at 60 °C, modified graphite exhibits a higher removal rate.

### 3.5. Effect of pH on Adsorption Performance

**Figure 7:**
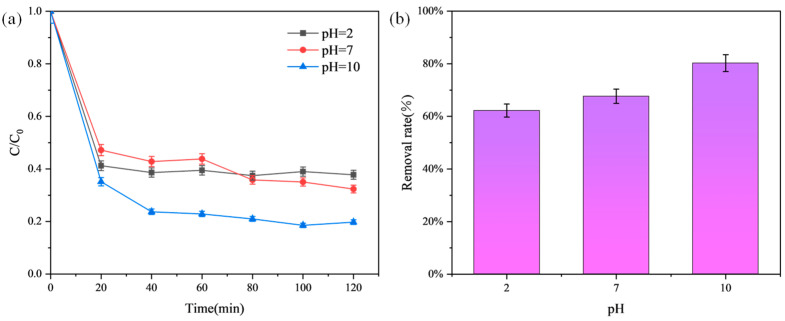
Effect of pH on MB adsorption by the optimal adsorbent. (**a**) Adsorption curves of the adsorbent for MB at different pH levels; (**b**) Removal efficiency of MB by the adsorbent at different pH levels after 120 min.

An 8 mg prepared adsorbent was placed in small glass vials containing 5 mL of 50 mg/L MB solution. The pH of the solution was adjusted using HCl (0.1 M) and NaOH (0.1 M) to pH values of 2, 7, and 10. The solutions were stirred at a magnetic stirring speed of 550 rpm and a temperature of 25 °C for 120 min to reach adsorption equilibrium, to investigate the effect of pH on MB removal. Figure 7 shows the adsorption effect of modified graphite on MB at different pH levels.

When 8 mg of adsorbent was added at pH values of 2, 7, and 10, it was evident that modified graphite exhibited a higher removal rate at pH 10. This is because the pH value of the solution affects not only the dissociation of the adsorbent but also the ionization state of the adsorbate in the aqueous solution. More importantly, the point of zero charge (PZC) of the adsorbent is approximately 9.7, and the pKa of MB is approximately 3.8~4. In more detail, at pH > 10, methylene blue exists almost entirely in a neutral form (deprotonation), and the pH of the organic dye solution is higher than that of PZC, and the surface is negatively charged. There is no significant electrostatic repulsion between neutral methylene blue and negatively charged modified graphite adsorbent surfaces, and the adsorption effect may be significantly improved. Therefore, a pH of 10 is more favorable for adsorption.

### 3.6. MB Adsorption

#### 3.6.1. MB Isothermal Adsorption of Modified Graphite

Adsorption isotherms are used to evaluate the suitability of the adsorption process and to assess the maximum adsorption capacity of the adsorbent material. Fitting isotherm models to experimental data provides information about the adsorption properties. There are several equations for adsorption equilibrium data, among which the commonly used surface adsorption models for single-solute systems are the Langmuir and Freundlich isotherm models.

**Figure 8:**
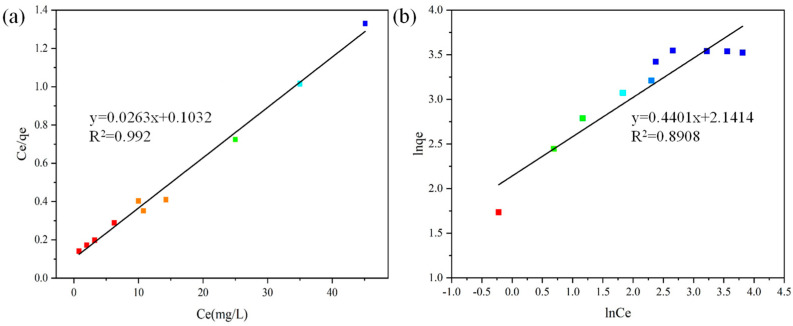
(**a**) Langmuir isotherm adsorption linear simulation plot; (**b**) Freundlich isotherm simulation plot.

According to Equation (3), the corresponding Langmuir isotherm adsorption data were calculated, as shown in Table 3.

(3) $$\frac{C_{e}}{q_{e}}=\frac{1}{q_{m}K_{L}}+\frac{C_{e}}{q_{m}}$$

where $q_{e}$ is the amount of MB adsorbed at equilibrium (mg/g), $q_{m}$ is the maximum monolayer coverage capacity (mg/g), $K_{L}$ is the Langmuir constant (L/mg), and $C_{e}$ is the equilibrium concentration of MB (mg/L). The linear relationship curve of MB surface adsorption on modified graphite was plotted under the conditions of 1.6 mg/mL adsorbent dosage, solution pH = 10, contact time of 120 min, and solution temperature of 25 °C, as shown in Figure 8a.

The Freundlich isotherm model describes heterogeneous multilayer adsorption behavior and can be expressed as Equation (4).

(4) $$lnq_{e}=lnK_{F}+1/n_{F}lnC_{e}$$

where $q_{e}$ is the amount of MB adsorbed at equilibrium (mg/g); $C_{e}$ is the equilibrium concentration of MB (mg/L); and *K_F_* and *n_F_* are Freundlich constants related to adsorption capacity and adsorption intensity, respectively. The corresponding Freundlich isotherm adsorption data calculated according to Equation (4) are shown in Table 4, and the corresponding linear relationship curve is plotted as shown in Figure 8b.

As shown in Table 3 and Table 4, the R^2^ value of the Langmuir model (0.9920) is significantly higher than that of the Freundlich model (0.8908). Additionally, the maximum adsorption capacity of MB (q_m_) calculated using the Langmuir model closely matches the actual experimental data, indicating that the experimental data of MB adsorption on modified graphite align better with the Langmuir model than the Freundlich isotherm model for the adsorption of organic dyes on fly ash-based coal graphite. This analysis suggests that MB adsorption on modified graphite conforms to the Langmuir isotherm model, which implies monolayer adsorption on the adsorbent surface. The Langmuir isotherm model assumes monolayer adsorption of the adsorbate on a homogeneous adsorbent surface with uniform binding sites. In contrast, the Freundlich model acknowledges multilayer adsorption processes on heterogeneous adsorption sites. The correlation coefficient (R^2^) and q_m_ values are more consistent with the experimental data, thus confirming the applicability of the Langmuir model.

The essential characteristics of the Langmuir isotherm can be represented by the dimensionless equilibrium parameter R_L_ = 1/(1 + bC_0_), where b is the Langmuir constant and C_0_ is the initial dye MB concentration (mg/L). In this experiment, the R_L_ values ranged between 0 and 1, indicating favorable adsorption of MB by modified graphite.

#### 3.6.2. MB Adsorption Kinetics of Modified Graphite

**Figure 9:**
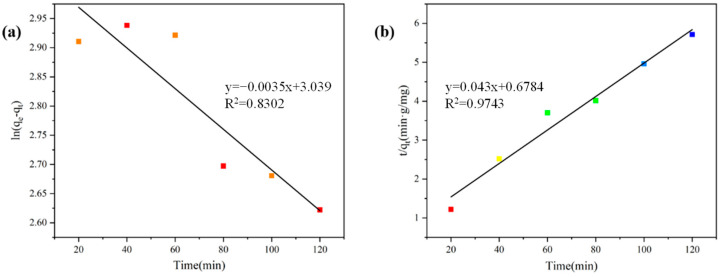
Adsorption kinetics of MB on modified graphite: pseudo-first-order adsorption kinetics model (**a**); pseudo-second-order adsorption kinetics model (**b**).

From Figure 9 and Table 5, it is evident that the adsorption data fit well with the pseudo-second-order model (R^2^ = 0.9743), indicating that chemisorption is the main rate-controlling step for the adsorption of MB by modified graphite [25,26].

#### 3.6.3. MB Adsorption Thermodynamics of Modified Graphite

The thermodynamic parameters and their mutual relationship between each other are represented in Equations (5)–(7).

(5) $$lnK=\frac{\Delta S}{R}-\frac{\Delta H}{RT}$$

(6) ΔG=−RTlnK

(7) $$K=\frac{q_{e}}{c_{e}}$$

where *R* (8.314 J mol^−1^ K^−1^) and *T* (K) are the gas constant and absolute temperature, respectively. *K* is the equilibrium constant which can be obtained via Equation (7). Ce (mg/L) and qe (mg/g) are the equilibrium concentration of adsorbate in an aqueous solution and the equilibrium uptake amount of the adsorbate, respectively. Δ*S* and Δ*H* can be obtained from the intercept and slope, respectively, from plotting *lnK* against *1/T*.

The summary of thermodynamic parameters calculated according to Equations (1)–(5) to (1)–(7) is shown in Table 6. As the temperature increases, Δ*G* is negative, which indicates that the adsorption of MB by modified graphite is a spontaneous process. The adsorption of MB on modified graphite is based on the endothermic process with positive Δ*H* = +57.1840 kJ·mol^−1^, indicating that the adsorption favors high-temperature conditions in improving the adsorption capacity. Δ*S* was determined to be 210.5600 J mol^−1^·K^−1^, indicating an increase in the degree of freedom of adsorbed material during surface adsorption. In summary, the adsorption of MB on modified graphite is a spontaneous endothermic reaction.

## 4. Conclusions

In this study, coal-based graphite was used as raw material, potassium permanganate as an oxidant, and mixed acid composed of nitric acid and phosphoric acid as an intercalation agent to prepare an adsorbent. The structure of coal-based graphite was regulated by high-temperature heat treatment, and the effect of adsorption performance of porous carbon-based adsorption material in organic dyes was investigated. It has been confirmed that high-temperature treatment can significantly change the microstructure of modified graphite–porous carbon-based material, increase its specific surface area, and introduce more active sites; particularly, the removal rate of MB can surpass 85%. In addition, an ultrafast and high-temperature furnace is superior to a vacuum tube furnace for the high-temperature treatment. An ultrafast and high-temperature furnace can vaporize the interlayer compounds of graphite instantaneously to achieve the expansion effect. The MB removal rate of modified porous carbon-based material prepared by this process can reach 91.89%. The fitting analysis of kinetic, thermodynamic, and isothermal adsorption models further revealed that the adsorption process was mainly controlled by chemisorption, which was consistent with the pseudo-first-order model. Thermodynamic data showed that the adsorption of MB on porous carbon-based adsorption material was a spontaneous endothermic reaction, and the adsorption process followed the Langmuir isothermal model, indicating the possibility of monolayer adsorption.

## Figures and Tables

**Table 1:** Raw material sample ratio and experimental conditions.

Sample	Reaction Time/h	Temperature/°C	Coal-Based Graphite/g	Phosphoric Acid/mL	Nitric Acid/mL	Potassium Permanganate/g
A	1	30	2	20	12	0.8
B	2	30	2	20	12	0.8

**Table 2:** The control temperature and equipment of samples.

Sample	Sample Number	Reaction Time/h	Temperature/°C	Equipment
A	A-1	1	600	UFTF
	A-2		800	UFTF
	A-3		800	VTF
	A-4		1000	UFTF
	A-5		1200	UFTF
	A-6		1400	UFTF
B	B-7	2	600	UFTF
	B-8		800	UFTF
	B-9		800	VTF
	B-10		1000	UFTF
	B-11		1200	UFTF
	B-12		1400	UFTF

**Table 3:** Langmuir isotherm adsorption-related data.

Kinetic Model	Parameters	MB
Langmuir	q_m_ (mg/g) calculated	38.02
	K_L_ (L mg^−1^)	2.47
	R^2^	0.992
	R_L_	0.0041~0.0397

**Table 4:** Freundlich isotherm adsorption-related data.

Kinetic Model	Parameters	MB
Freundlich	K_F_ (mg g^−1^) × (L mg^−1^)^1/n^	8.8113
	1/n_F_	0.4401
	R^2^	0.8908

**Table 5:** Pseudo-first-order, pseudo-second-order, and intraparticle diffusion dynamics model equations.

Kinetic Model	Parameters	MB
Pseudo-First-Order	q_e_ (mg/g) calculated	1.004
	q_exp_(mg/g)	34.7625
	k_1_ (min^−1^)	0.0081
	R^2^	0.8302
Pseudo-Second-Order	q_e_ (mg/g) calculated	23.26
	q_exp_ (mg/g)	34.7625
	k_2_ (g/mg min)	0.004
	R^2^	0.9743

**Table 6:** Thermodynamic parameters for the adsorption of MB on modified graphite.

Adsorbent	ΔH (kJ mol^−1^)	ΔS (J K^−1^ mol^−1^)	ΔG (kJ mol^−1^)		
			283 K	298 K	318 K
Modified graphite	57.1840	210.5600	−2.4045	−5.5629	−9.7741

## Data Availability

The original contributions presented in the study are included in the article, further inquiries can be directed to the corresponding author.
